# The Calibration of dc Voltage Standards at NIST

**DOI:** 10.6028/jres.095.027

**Published:** 1990

**Authors:** Bruce F. Field

**Affiliations:** National Institute of Standards and Technology, Gaithersburg, MD 20899

**Keywords:** calibration services, dc volt, standard cells, voltage standards, Zener diode standards

## Abstract

This document describes the procedures used at NIST to calibrate dc voltage standards in terms of the NIST volt. Three calibration services are offered by the Electricity Division: Regular Calibration Service (RCS) of client standard cells at NIST; the Volt Transfer Program (VTP) a process to determine the difference between the NIST volt and the volt as maintained by a group of standard cells in a client laboratory; and the calibration of client solid-state dc voltage standards at NIST. The operational procedures used to compare these voltage standards to NIST voltage standards and to maintain the NIST volt via the ac Josephson effect are discussed.

## 1. Introduction

To maintain a “practical” unit of electromotive force (emf), that is, a representation of the volt as defined in the International System of Units (SI), standards laboratories may use one or more groups of electrochemical cells called standard cells^1^. Such cells are used as reference standards against which the emf of other cells or the electric potential difference of systems are compared. Typically, saturated cadmium-sulfate-type (Weston) cells are used when high accuracy, one part per million (1 ppm) or better, is required in maintaining a unit traceable to NIST. However, dc voltage standards based on solid-state devices are now available with stabilities approaching 1 ppm/yr. Such standards can be used to maintain and disseminate (transfer) a laboratory unit of voltage with accuracies approaching those obtainable with good saturated standard cells.

Standard cells are electrochemical systems composed of two dissimilar electrodes immersed in an electrolytic solution [1]. They are not intended to supply electric current and, therefore, are of different design from those electrochemical systems (e.g., storage batteries) which are intended for such purpose. The stability of the emf of the cell depends on the chemical equilibrium within the cell. Although the emf of a cell is highly reproducible and generally exhibits a fairly constant emf, it must be periodically recalibrated to eliminate the effects of long term drift. The frequency with which recalibrations are required is a function of the client’s accuracy requirement, the number of cells used to maintain their volt, and the stability of their individual cells. This recalibration interval must be determined by the client laboratory based on their accuracy requirements and cannot be specified by NIST.

Presently available solid-state voltage standards contain at least one (and sometimes more) Zener-diode reference devices each of which develop an electric potential difference that is scaled using internal resistive dividers and low-noise amplifiers to produce a number of fixed-value output voltages in the range from 1 to 10 V. Outputs are typically provided at 1, 1.018, and 10 V, although the unscaled Zener voltage of about 7 V is also occasionally provided. These standards usually have their best stability at the Zener voltage (if available) or at 10 V rather than at the 1.018-V level of standard cells, as the scaling process from 10 to 1.018 V introduces additional uncertainty.

Calibration of dc voltage standards in terms of the NIST volt is accomplished via three services offered by the Electricity Division of the NIST. These calibration services are described briefly below, while the measurement approach, operational procedures, maintenance of the volt, and calibration uncertainties are described more fully in sections 2–5 of the paper. Additional details about the calibration services and their measurement procedures can be found in references [2] and [3].

### 1.1 Regular Calibration of Standard Cells

Clients may send standard cells to NIST for direct comparison to NIST cells which are in turn calibrated in terms of the NIST volt. Upon receipt of the standard, its cell emfs are compared directly to the NIST voltage standards on a daily basis and, after the emfs have stabilized, 10 additional comparisons are made. The averages of the cell emfs obtained from these comparisons are taken as the measured values of the cells. At the conclusion of the test a report is issued with a statement of the cell emf values and the estimated uncertainties for the emf values. These uncertainties apply only to the measurements made while the cells were at NIST. Since no additional components of uncertainty are included for transportation effects, long term drifts of the emfs, or measurement errors in the client’s laboratory, such uncertainty components must be evaluated and included by the client as part of a complete analysis of the uncertainty of the standard.

### 1.2 Volt Transfer Program for Standard Cells

The Volt Transfer Program (VTP) is a measurement assurance program (MAP) designed to determine the difference between the unit of voltage maintained by a client laboratory using standard cells and the NIST volt. In this program, a group of NIST-owned cells in a thermoregulated enclosure is measured at NIST, shipped to the client laboratory, compared by the client to their reference cell group, and finally returned to NIST for concluding measurements. The measurements at the client laboratory are made using the client’s measuring apparatus but using procedures specified by NIST. As data are taken they are transmitted to NIST and reduced and reviewed by NIST. At the conclusion of the test, a report is issued giving the difference between the client unit of voltage and the NIST volt as observed during the time of the test, along with an uncertainty for the difference. In this experiment, the uncertainty caused by the transportation of the standard cells is estimated and included in the final uncertainty.

### 1.3 Calibration of Solid-State Standards

Solid-state voltage standards to be calibrated by NIST must be calibrated at NIST (no MAP is presently available) and they must be complete instruments that continuously produce one or more stable voltages. When sent to NIST, these standards are compared indirectly to NIST standard cells which are in turn calibrated in terms of the NIST volt. NIST uses a specialized measuring/scaling system that automatically compares the output of any arbitrary voltage source within the voltage range of 1 to 10 V to a 1.018-V reference standard. Ten comparisons are made (one each working day) over a 2 week period as a minimum. At the conclusion of the test, a report is issued stating the values of the voltage outputs of the standard and their estimated uncertainties while the standard was at NIST. No additional uncertainty is included for transportation effects, long term drifts of the outputs, or measurement errors in the client’s laboratory.

## 2. Measurement Approach

### 2.1 Standard Cell Voltage Difference Measurements

**Figure 1:**
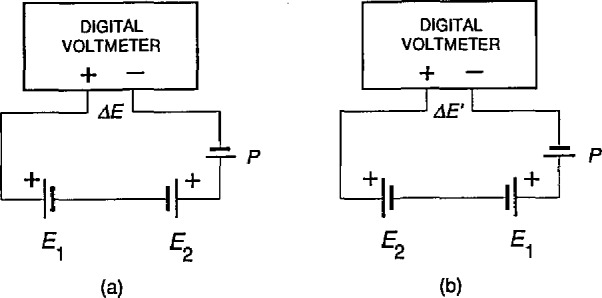
Simplified schematic of the measuring circuit used for series-opposition comparison of standard cells, (a) The “normal” measurement (b) The “reversed” measurement with the cells interchanged to eliminate the constant positional effect denoted as a fixed emf *P*.

All cells at NIST are calibrated by comparing them directly to NIST-owned standard cells using a series opposition method and redundant measurement designs [5]. Figure 1(a) shows a simplified schematic of the measuring circuit used for the series opposition measurement method. Two cells (one of known value) are connected in series opposition and the small voltage difference is read using a digital voltmeter (DVM). However, in any real measurement circuit there will be unwanted spurious emfs as illustrated by the emf source P in figure 1(a). In general these spurious emfs can be classified into two categories:

1. Those emfs that remain constant, or relatively so, in relation to the interval over which a complete set of measurements is made.
2. Those emfs that vary rapidly (referenced to the interval over which a complete set of measurements is made).

If the emfs are of the second type they will have the effect of decreasing the precision of the process. On the other hand, if they are of the first type they will have the effect of introducing a systematic error into the measurement result such that

$$\Delta E=E_{1}-E_{2}+P$$

where *E*_1_ and *E*_2_ are the emfs of the two cells being compared and *P* is the constant, and as yet unknown, emf. It is possible to estimate *P* by reversing the position of the cells in the circuit and taking a second measurement Δ*E′* as shown in figure 1(b), where

$$\Delta E\prime =E_{2}-E_{1}+P.$$

Taking the difference between the two expressions gives

$$\Delta E-\Delta E\prime =2(E_{1}-E_{2}),$$

thus yielding an estimate of *E*_1_*—E*_2_ free of *P*. The pair of measurements are said to be “left-right” balanced. That is, if there is a positional effect in the circuit it is balanced out of the final result. This technique is analogous to that used to eliminate the inequality of balance arms in precision weighing on a two-pan “equal-arm” balance. In order to identify the cell positions from the operational point of view, they are frequently designated as left and right relative to the input terminals of the measuring instrument. In practice more complex measurement patterns, i.e., redundant measurement designs, are used for intercomparing standard cells, see section 2.3.

### 2.2 Standard Cell Measurement Apparatus

**Figure 2:**
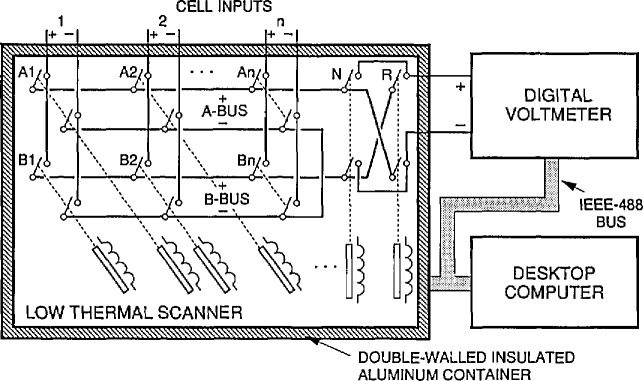
A diagram of the automated cell comparison systems showing some details of the low-thermal scanner. The scanner and digital voltmeter are controlled by a desktop computer.

Two fully-automated voltage measuring systems are currently in use at NIST for all cell comparisons. Except for the switching systems, they are identical; one system (VTP) has a low-thermal-emf scanner with 80 voltage (cell) inputs and the second system (RCS) has a low-thermal scanner with 300 inputs. Figure 2 is a simplified diagram that generally represents both systems (the scanners vary in minor details). In addition, both systems contain a high resolution 6-1/2 digit voltmeter, a desktop computer, and a shared hard disk system (not shown).

The scanner permits selection of any pair of standard cells and connects them, in series opposition, to a digital voltmeter. The scanner effectively consists of two scanners: one cell is connected to the “A-bus” (see fig. 2) and a second cell is connected to the “B-bus.” The bus (cell) negative terminals are connected together and the positive terminals are connected to the DVM through additional reversing switches. The switch assemblies are specially modified mechanical crossbar-matrix switches that use phosphor bronze springs and gold contacts and are actuated by magnetically latching solenoids. The use of latching solenoids reduces the heat input from the solenoid to the switch assembly (and thus the thermal emfs) when the switches are actuated. As the switches have relatively large thermal emfs when placed unprotected in the laboratory, they are mounted in a nested-pair of heavy-walled aluminum chambers with insulation between them. Additionally, thermal shunts are tied to the incoming leads. These thermal management techniques reduce the total thermal emfs in the scanner to typically 15 and 30 nV, respectively, for the 80- and 300-input switches. The difference in the thermal emfs of the two scanners is attributed to small deliberate differences in the construction of the switches to accommodate different numbers of cells. The scanners are controlled through an instrumentation bus [6].

The voltmeters were selected (and individually tested) for low input bias current and low random measurement error. Since the saturated standard cell emf stability depends on an electrochemical equilibrium within the cell, small charging or discharging currents that are present at the input of the DVM will produce changes in the cell emf that may be significant. The voltmeters used at NIST have been tested and determined to have an input bias current of 12 pA which contributes a negligible error to the measurement. A total measurement time of 18 s for each measurement is used by averaging six 3-s duration DVM readings which results in a typical single measurement standard deviation of 0.034 *μ*V. In additional experiments, identical cell comparisons were made using both the DVM and classical potentiometers and the measurement results agreed well within 0.03 *μ*V, the combined uncertainties of the instruments.

### 2.3 Redundant Measurement Designs

As used in this context, redundant measurement designs are a specified sequence of measurements used to compare pairs of standards. The measurement sequence is chosen to provide an optimum amount of information with as few measurements as possible. For the NIST measurements, the parameters that are estimated by the designs are: the difference of each cell emf from the reference group mean emf, the within-day standard deviation (the process precision), and the left-right (positional) effect in the measuring system. In addition, the designs provide diagnostic information about possible errors or problems with the measuring system. The within-day standard deviation permits laboratory personnel to estimate the quality of the measurements immediately and the redundant measurements allow them to eliminate anomalous observations mathematically from the measurement design and re-compute the cell emfs with little loss in accuracy.

Two specific designs are generally used at NIST, one for comparison of two groups of four cells, and one for comparison of one group of six cells to one group of four cells. In both cases a “full” design is used where all possible pair differences that involve cells from different groups are measured (16 and 24 measurements respectively). For other larger groups of cells, some observations may be eliminated from the “full” design. The cell emfs are estimated from the cell difference measurements by solving the over-determined set of equations using the least-squares method and including the constraint that the mean emf of a given group of cells (i.e., the reference group) is known. (When comparing client cells to NIST cells, the NIST cells are specified as the reference group.) This results in an assignment of individual emf values to all the cells in terms of the mean emf of the reference group.

When a well-designed classical potentiometer is used as the cell comparison instrument the left-right effect *P* does not vary significantly over the time of the measurement design and it is sufficient to require that the total design be left-right balanced, i.e., each cell appears on the left side and right side of the measuring system an equal number of times irrespective of their order of appearance in the design. Digital voltmeters, however, have been found to introduce a small time-varying dc offset that must be eliminated by immediately reading every cell pair a second time with the polarity of the input reversed. The algebraic difference of these two measurements divided by two is considered to be a single measure of the cell difference with the DVM offset eliminated.

### 2.4 Temperature Measurements

All saturated standard cells exhibit an emf change with temperature and must be maintained at a constant temperature. Cells sent to NIST for calibration are either housed in their own portable thermoregulated enclosures, or are immersed in NIST-provided constant-temperature oil baths. Thermoregulated enclosures generally contain a temperature sensing element which can be used to monitor the small temperature variations (usually less than 0.01 °C) within the cell enclosure. Typical devices are mercury-in-glass thermometers, thermistor bridges, and platinum resistance thermometers. NIST follows manufacturers’ recommended procedures for monitoring the temperature using these devices. The temperature scale embodied in the temperature device is generally taken as correct, primarily because accurate knowledge of the temperature is unnecessary. Cell emfs are corrected only for small changes in temperature, referenced to a nominal temperature as established by the temperature device.

For cells housed in the NIST oil baths, calibrated platinum resistance thermometers and an ac resistance thermometer bridge are used to measure the temperature. The temperature of the oil bath is stable and uniform to at least 0.001 °C. The estimated uncertainty of the temperature measurement with respect to the International Temperature Scale of 1990 (ITS-90) is 0.005 °C (3 standard deviation estimate), and includes uncertainty components for the power dissipation in the thermometer, the determination of the triple point of water, and the drift of the calibration constants between calibrations.

Cell emfs are corrected for small day-to-day temperature changes by monitoring the temperature as described above and applying an emf correction based on the International (or Wolff) Temperature Formula [11]:

$$E_{t}=E_{20}-0.0000460(t-20)-0.000000950(t-20{)}^{2}+0.000000010(t-20{)}^{3}$$

where *E*_t_ is the emf in volts at temperature *t* and *E*_20_ is the emf at 20 °C. This equation is used to correct the cell emfs to any arbitrary nominal temperature by computing the correction for the actual temperature with respect to 20 °C and subtracting the correction for the nominal temperature with respect to 20 °C. The formula is an approximation and is not exact for all cells although it provides reasonable accuracy (better than 0.1 *μ*V) if the total correction is less than 1 *μ*V.

### 2.5 Solid-State Voltage Reference Measurements

Solid-state voltage standards are calibrated at NIST using a specially designed measurement system (denoted “ZCS”). It employs a Potentiometric method allowing voltage measurements to be made without loading the standard. (An earlier version of this system is described in reference [7]). The system is a fully-automated scaling device for comparing arbitrary voltage standards to 1.018-V standard cells. A three step procedure is used to make a measurement of an unknown voltage: 1) the system is self-calibrated using a group of standard cells of known emf, 2) the unknown solid-state standards are compared to the calibrated system, and 3) the system is self-calibrated again to make sure that no significant drifts in the system have occurred.

**Figure 3:**
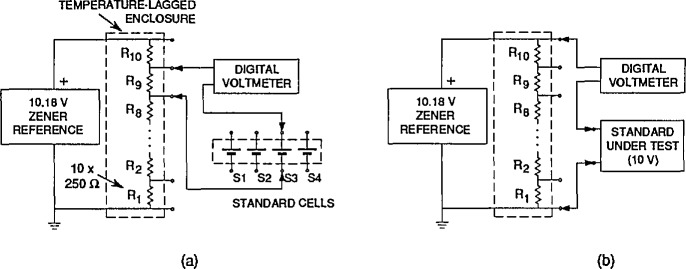
A simplified diagram of the “ZCS” calibration system used to calibrate solid-state standards, (a) The system arranged for self-calibration of the 1.018-V voltage drops across each of the 10 resistors, (b) The system configured for calibration of an unknown standard-under-test of approximately 10 V.

Figures 3a and 3b show a simplified diagram of the measuring system configured for self-calibration and for measurement of unknown solid-state standards respectively. A modified 10.18-V Zener reference is permanently connected to a resistive divider; this combination (the Transfer Reference) is considered to be a stable voltage source with 10 series-connected 1.018-V outputs. The Transfer Reference is calibrated by comparing the voltage drop across each resistor in turn to the emf of each cell in a four cell group, measuring the microvolt-level difference voltages with the digital voltmeter for a total of 40 measurements. (Not shown in fig. 3 is a low-thermal scanner, similar to the ones described previously, which permits selecting the various voltage differences. Also not shown is the desktop computer and the shared hard disk system.) After calibration, the Transfer Reference is used as a calibrated source producing 10 voltages from 0 to 10.18 V in steps of 1.018 V by referencing each output tap to the bottom tap. Then (as shown in fig. 3b) the output voltage developed across *N* of the divider resistors is compared to the voltage of the unknown standard-under-test using the DVM. *N* is chosen to minimize the magnitude of the DVM reading. After all the standards under test have been measured, a second self-calibration of the Transfer Reference is performed to reduce any error caused by its drift during the measurements.

The final value for each solid-state standard under test is computed by correcting the measured difference between the standard-under-test and the Transfer Reference for the gain error of the DVM, and adding to this the value of the appropriate tap of the Transfer Reference. The calibrated values of the Transfer Reference are determined using a least-squares analysis of the redundant measurements that compared the 10 outputs of the Reference to the four-cell reference standard cell group. The mean of the “before” and “after” calibrations is used.

With this system the DVM is used to read only a fraction of the voltage of the standard-under-test which reduces the contribution of the DVM uncertainty to the overall measurement uncertainty. When comparing the standard-under-test to the Transfer Reference (with voltage taps at 1.018 V increments) the maximum possible reading required of the DVM is 0.509 V. Thus,

$$U=\frac{V_{z}-(N)1.018}{V_{z}}U_{\mathrm{DVM}}$$

where *U*_DVM_ is the DVM uncertainty expressed as a percentage of reading, *V*_z_ is the voltage of the solid-state standard, *N* is the number of resistors, and *U is* the final DVM uncertainty as a percentage of *V*_z_. For a worst case of *V*_z_=5.6 V, *U*=0.09 *u*_dvm_, and for *V*_z_=10 V, *U*=0.02 *U*_DVM_. If, for example, the DVM were calibrated to an accuracy of 1 ppm of reading (not an unreasonable assumption) the DVM error contribution at 10 V would be 0.02 ppm.

The two largest contributions to the DVM uncertainty are the linearity error of the DVM and the gain error. (All DVM measurements are combined measurements of a “normal” and a “reversed” DVM reading to eliminate the DVM offset error.) The linearity of the DVM is checked periodically using an independent calibration system. The gain of the 1 V range of the DVM (the one used for all high accuracy measurements) is calibrated as part of step 2 of the measurement procedure by making two 1.018-V measurements of the voltages developed across two resistors in the Transfer Reference. Two measurements are made to evaluate the effect of measuring voltages with the DVM at ground and above ground. These gain calibrations are used by the system as previously described to correct the DVM readings to reduce the overall measurement uncertainty.

The DVM is used on the 0.1-V range for improved resolution in the comparisons of 1.018-V solid-state standards and of the reference standard cells to the Transfer Reference. The gain error of the 0.1-V range is not measured daily by the system but, the largest expected voltage difference for these comparisons is only 200 *μ*V. Thus, for an overall measurement error of 0.005 ppm (of 1.018 V), the gain need only be known to 250 ppm. The gain of the 0.1-V range is periodically checked using external standards to ensure that it is within this limit.

## 3. Maintenance of the NIST Volt

By international agreement, starting on January 1,1990, a highly accurate representation of the volt based on a new constant for the Josephson effect has come into effect worldwide [8]. This representation is defined in terms of the Josephson constant *K*_J_, the frequency-to-voltage quotient of the ac Josephson effect in superconductors. The value of *K*_J_ used for maintaining the volt representation at NIST is 483 597.9 GHz/V. This value is believed to define a volt representation consistent with the SI volt to within 0.4 ppm (one standard deviation estimate). The operational procedures and apparatus used to maintain the NIST volt are described briefly below; additional details can be found in reference [9].

### 3.1 The ac Josephson Effect

When two weakly coupled superconductors are irradiated with microwave energy, the assembly (a Josephson junction) can be used to produce a number of precise voltages, *U*_J_(n), described by the following equation:

$$U_{J}(n)=nf/K_{J},$$

where *n* is an integer, *f* is the frequency of the irradiating microwave energy, and *K*_J_ is as described above. A variety of experimental tests (for material dependence, temperature dependence, etc.) and theoretical investigations of the Josephson relation have been made which indicate that this equation is exact [10].

### 3.2 The Josephson Array

Arrays consisting of from 1500 to 2076 Joseph-son junctions are used to produce a total voltage of up to 1.2 V [11,12]. These arrays do not require control of the bias currents for the individual junctions as was the case in the past because they use constant-voltage steps which cross the zero current axis of the junction I-V curve. This arrangement allows a large array of junctions to share a common current bias at or near zero. The arrays are fabricated using niobium and lead alloys and are stable at room temperature.

### 3.3 Measurement Apparatus

**Figure 4:**
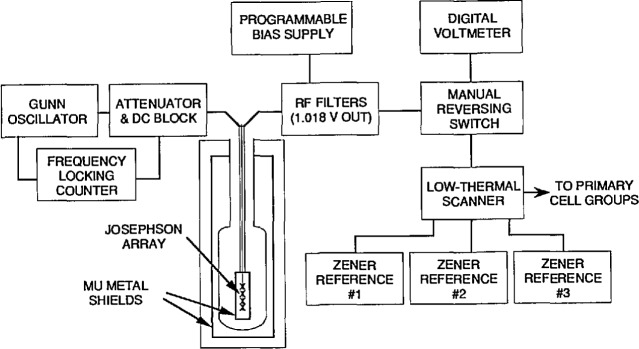
Diagram of the voltage measurement system based on the Josephson array.

Figure 4 is a simplified diagram of the Josephson array measurement apparatus. The microwave radiation is supplied to the Josephson array by a 60-mW Gunn diode oscillator at 94 GHz which is frequency-stabilized by a frequency-locking counter containing a quartz-crystal oscillator. The short term frequency stability of the microwave radiation (15 min) is about 1 part in 10^9^. The frequency is measured by the frequency counter with a resolution of 1 part in 10^10^. The accuracy of the counter time base is regularly checked against the U.S. frequency standard by comparing the counter time base to a 100 kHz high-stability oscillator which is simultaneously compared to the signal from WWVB using a VLF comparator.

Also shown in figure 4 is the dc measurement apparatus used to compare the array voltage at 1.018 V to a 1.018 V Zener reference standard. The bias source supplies a small current to force the Josephson array to operate at the desired step. RF filters are incorporated at the top of its probe to prevent externally generated noise from disturbing the array. The manual reversing switch contains multiple switches to perform two functions: reversing the polarity of the signal to the DVM, and reversing the polarity of the Zener reference (selected by the scanner) to match the polarity of the array. Thus, to measure the voltage of a Zener reference, the array is adjusted to produce a voltage nearly equal to the Zener reference by (a) adjustment of the bias current and microwave power to select a suitable voltage step, and (b) adjustment of the microwave frequency to fine-tune the step voltage. The step is observed on an oscilloscope (not shown in the figure) to check for any abnormalities. The difference between the array and Zener reference is then measured by averaging several readings (*E*_1_) of the digital voltmeter. The polarity of the DVM is reversed and several more readings (*E*_2_) are taken to eliminate offsets in the digital voltmeter. The bias current and microwave power are then adjusted to produce an array voltage of —1.018 V and the polarity of the Zener reference is reversed to match the array voltage. Two more sets of readings (*E*_3_ and *E*_4_) are taken with the DVM in its normal and reversed polarities respectively. This action is required to eliminate thermal emfs in the leads from the array to the reversing switches. The Zener voltage is calculated as (*E*_1_*—E*_2_*—E*_3_*+E*_4_)4*+nf/K*_J_ where *n* is the integer step number, *f* is the microwave frequency, and *K*_J_ is as defined above. This measurement sequence is repeated five times with a typical standard deviation of 0.009 *μ*V for the five measurements, and takes about 12 min.

A final check is made on the thermal emfs in the leads from the Zener reference to the manual reversing switch by replacing the Zener reference with a short and adjusting the array to operate on the zero-voltage-step with the microwave power set to zero. The same measurement sequence is run as for the 1.018-V measurement. The residual thermal emfs thus determined are subtracted from the 1.018-V measurements.

## 4. Operational Procedures

Although it is theoretically possible to compare all 1.018-V client standards directly to the Joseph-son array, it would be inconvenient to do so as the Josephson measuring system is still partially manually controlled at present and the large number of measurements required each day could not be accomplished with just one measuring system. Instead, a hierarchical path of cell groups has been established to enable client standards to be calibrated in terms of the Josephson array.

**Figure 5:**
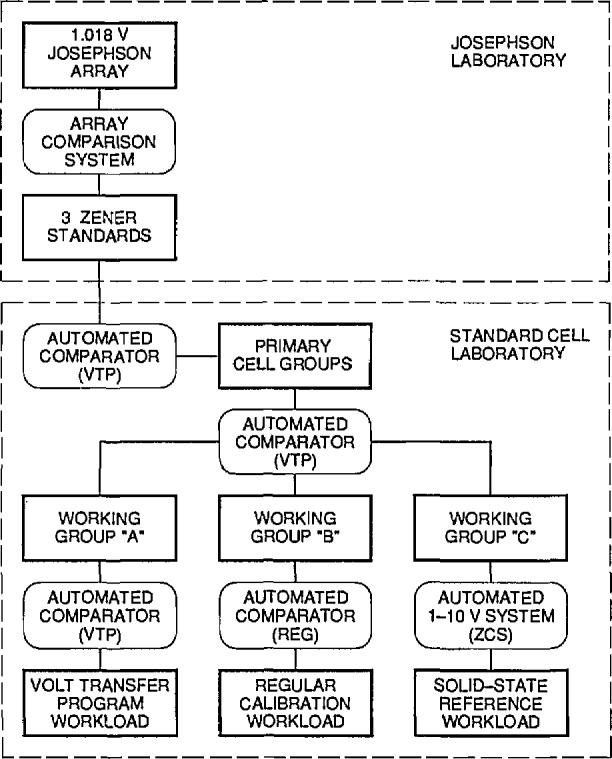
Plan of the voltage traceability process from the Josephson array to the workload for the three calibration services.

Figure 5 shows the path of measurements from the Josephson array to client standards to be calibrated and the three measuring systems used (VTP, RCS, and ZCS). The details of these measuring systems have been described above (in sees. 2 and 3).

### 4.1 Calibration of the Laboratory Primary Cells

Comparison of the Josephson array to groups of cells considered to be “primary” cells (Josephson measurements) are made at approximately weekly intervals. Modular switches (using components similar to the previously described low-thermal scanners) permit comparing standards in the Josephson laboratory to cells situated in the Volt Facility (adjacent to the Josephson laboratory) with very small thermal emfs being introduced in the measurements. The weekly comparisons of the three Zener standards in the Josephson laboratory to the primary cells consist of three steps. 1) The three Zener standards are compared to the primary cells using redundant measurement designs and the VTP automated comparator. 2) The Zener standards are compared to the Josephson array and assigned voltage values. 3) The Zener standards are again compared to the primary cells. Every 4 weeks additional cell comparisons between the primary and working cell groups are made to investigate the possibility of new systematic errors that may have developed in the measuring systems. This is designated a “cardinal” measurement.

**Figure 6:**
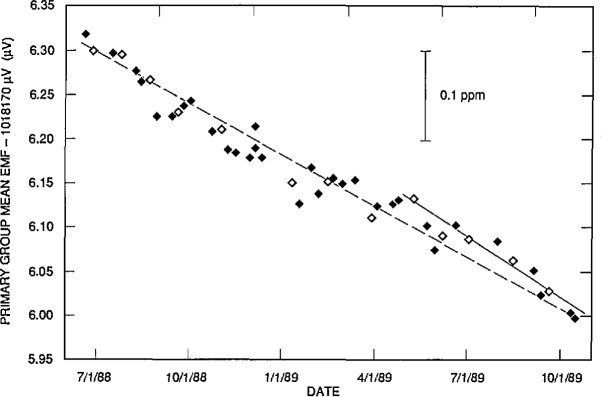
Josephson array measurements of the mean emf of the Primary Group. The open symbols indicate a cardinal measurement where new predictions for the cell values are computed. The meaning of the solid and dotted lines is described in the text.

Standard cell emfs drift with time so the use of a simple time invariant model for the cell emf can lead to unacceptably large step changes in the cell value each time the value is reassigned from the Josephson measurements. In addition, it is desirable to “average” several Josephson measurements to reduce the random error associated with the short term fluctuations of the cell emfs as well as with the measurements. Accordingly, we use a model for the primary cell group emfs that predicts a linear drift with time, and new model coefficients are calculated after every cardinal Josephson measurement. Figure 6 shows the typical behavior of the mean emf of the primary group plotted against time.

In addition, since the workload calibration process is continuous, it is not possible to wait for the next Josephson measurement before assigning values to client cells. Therefore, weekly values for the means of the emfs of the cell groups are predicted using a least-squares fit based on the last five or so cardinal measurements. (The solid line in fig. 6 is such a prediction.) The exact number of measurements chosen for the fit depends on (a) how well the cell emfs fit a linear model, and (b) the random scatter in the Josephson measurements of the cell emfs. The judgement and experience of the laboratory staff are used to determine the models. A fit to all the cardinal measurements in figure 6 (the broken line) has been included to emphasize that the cell emfs do not follow a straight line model for an extended time period; additional structure (possibly humidity related) appears in the data.

The three Josephson measurements made on “off-weeks” each month are used to check the prediction of the assignment of the primary cells. The collection of cell groups that constitute the primary groups changes as cells need replacement or enclosures need repair. In general, the primary groups consist of two or three groups of four to six cells each, with about 10 to 12 cells total. The primary cell groups also serve as a check standard for the Josephson measurements. If an individual Joseph-son measurement assigns values to the primary cells that are inconsistent with the predicted values (different by more than 0.07 ppm), all of the measurement systems are investigated to determine the source of the inconsistency. If the problem cannot be resolved, the Josephson measurement is usually repeated. In the exceptional circumstance where later Josephson measurements confirm the deviation from the predicted model, appropriate past workload data are corrected to reflect the change.

### 4.2 Calibration of Working Cell Groups

As previously discussed, in order to minimize the possibility of disturbing the primary cells, and because large numbers of standards are calibrated each day with three simultaneously operating measurement systems, the client cells cannot be compared directly to the primary cells. Instead, as shown in figure 5, the primary cells are compared to three working groups of cells once each day and then the working-group cells are compared to the client standards and NIST transport standards. The working-group cell emfs for each day are determined from the predicted values of the primary cells and the results of the comparisons of the working-group cells to the primary cells for that day. This process of using several working groups is feasible because only a small within-day uncertainty is introduced by the extra intercomparison measurements.

The constituents of the working groups (and the primary groups) are modified as necessary when cells show erratic behavior or temperature-regulated enclosures fail. (Historically substitutions have occurred every few years.) Changing cells occasionally is not a problem, particularly for the working groups, as no long-term history is required for operation of the system; the working groups are calibrated each day and must remain stable only throughout the day. The primary groups are required to be predictable for up to 1 month, this requires that candidate cells exhibit stable emf behavior for about 5 or 6 months before being eligible for use as primary cells. In addition to the cell groups considered to be primary and working, we have additional groups that are measured daily; these could substitute for the presently used groups.

### 4.3 Calibration of Client Saturated Standard Cells

Cells received for calibration may be subjected to a stabilization period before measurements are begun. The length of the stabilization is usually not longer than 4 weeks and depends on whether the cells were shipped to NIST under constant temperature control or not. If space is available on the measuring system, and cells have been shipped under temperature control, they are connected to the measuring system immediately. However, later review of the data generally results in these early measurements being discarded.

Three types of saturated standard cells are calibrated: cells intended for immersion in oil at 28 or 30 °C, and groups of cells in their own temperature-controlled enclosures. The first two types are placed in oil baths the temperatures of which are stable and uniform to at least 0.001 °C and are determined using NIST-owned platinum resistance thermometers. Temperature-controlled standard cell enclosures are tested under the following ambient conditions:

Temperature (23±1) °C
Relative humidity 50% or less

The operating temperature of the cells in temperature-regulated enclosures is determined using the temperature measuring device supplied with the enclosure. If the enclosure has a temperature indicating bridge, a NIST-owned null detector is used to make the readings to within the resolution of the bridge, usually 0.001 °C. A NIST or customer-owned platinum resistance thermometer may be used if requested. Temperature measurements are made each day before the cell emf measurements are started.

### 4.4 Calibration of Volt Transfer Program Standards

The Volt Transfer Program involves sending a NIST-owned transport voltage standard to the client laboratory. The transport cells are compared to NIST working standards at NIST before and after shipment to the client laboratory and to the client reference group of standard cells while at the client laboratory. The process is described in more detail below.

#### 4.4.1 Measurement of the Transport Standard at NIST

The transport standard consists of a commercial thermoregulated enclosure usually containing four shippable saturated standard cells, operating at an internal temperature of 30 or 32 °C. The temperature is measured using the internal temperature bridge and an external null detector to provide 0.001 °C resolution. Temperature corrections to the cell emfs are applied according to the discussion given in section 2. Transport standard cells are compared daily to Working Group A using redundant measurement designs and the VTP measurement system (see fig. 5). Before shipment to a client laboratory, the cell emfs are plotted and examined for stability. A minimum of 15 stable measurements are required before the enclosure will be shipped.

The transport standard is shipped in a special container designed to provide physical shock protection and ambient temperature lagging. It also contains an external power supply to provide regulated power to the transport standard to maintain the cells at a constant temperature while at NIST and the client laboratory. In addition, batteries within the supply power the transport standard during shipping for up to 24 h during normal conditions. Since the temperature control system cannot supply cooling to the enclosure, shipment to warmer locations is avoided during extremely hot weather to prevent the enclosure from overheating. The standard is shipped either via air freight or hand-carried by laboratory personnel. In the former case, the client laboratory personnel are notified of the flight time of the standard and are expected to provide transportation from the airport to their laboratory within the 24-h lifetime of the batteries.

#### 4.4.2 Comparison of the NIST Transport Standard to the Client Standard

Each laboratory participating in the Volt Transfer Program must identify a group of saturated standard cells that are considered to be the “laboratory reference group” and constitute the “laboratory volt.” The transport standard cells are compared to the cells of the laboratory reference using the normal laboratory measuring equipment and procedures with the exception that a NIST-specified measurement design must be used. NIST provides data sheets to record the measurement results. The data sheets are then immediately returned to NIST where the cell comparison data are reduced to determine the transport (and laboratory reference) cell emfs in terms of the client laboratory volt. Laboratories are required to make daily measurements and the results are reviewed by NIST to determine when sufficient data have been obtained to permit shipment of the standard back to NIST. A minimum of 10 measurement designs over 10 days is required for the transfer; however, the number is more typically in the range from 12 to 20. In addition, the within-day standard deviations and left-right components from the measurement designs are tested to see if they are in statistical control with respect to the expected values as determined from similar measurements at NIST and other laboratories.

#### 4.4.3 Final Report

**Figure 7:**
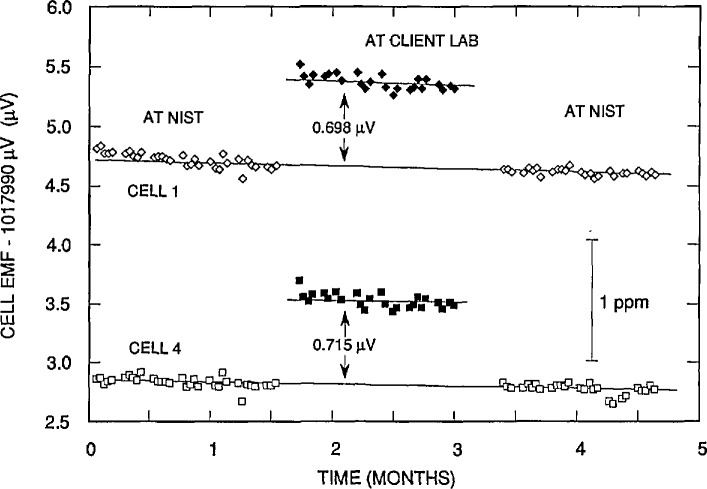
Typical measurement data for two of the four cells used for a Volt Transfer Program determination with a client laboratory.

Upon its return to NIST, the standard is again compared to Working Group A as before and, when the standard has stabilized and sufficient data has been taken, a final report is issued. Typical measurement data, the emfs of two of the four cells in a transport standard, are plotted in figure 7. Anomalous cell emf changes, typically caused by shipment or temperature problems, are eliminated from the final data analysis. (In fig. 7 the first measurement at the client laboratory for both cells appears slightly inconsistent with the succeeding data.) The data are also reviewed for abnormal temperature readings and to determine if they reasonably conform to a linear model.

Because the before and after NIST data do not usually exactly agree, a least-squares fit is made to them for each transport cell, and values are interpolated for each of the times (dates) the cell was compared to the client laboratory cells. V(LAB)-V(NIST) is determined using each cell in the transport enclosure by subtracting the NIST interpolated values of the transport cell from the respective client laboratory emf assignments, and taking the mean of these differences (0.698 and 0.715 *μ*V for the data of fig. 7). The difference V(LAB)-V(NIST) as determined from each of the four cells in the transport standard should agree, but on occasion there is disagreement caused by abnormal cell behavior, such as excessive drift, or poor recovery from a physical shock or a temperature disturbance. The judgement and experience of the laboratory staff are used to determine when to exclude a cell from the analysis or, on rare occasions, to use only the NIST data taken before or after the transfer, but not both.

### 4.5 Calibration of Solid-State Standards

Solid-state voltage references sent in for calibration are connected to measuring system ZCS as soon as they are received. These references are measured starting the day after their receipt without a stabilization period, since none is usually required. Most standards sent in for calibration provide some method for monitoring the temperature of the reference element(s) of the standard. For these standards, the temperature is measured before the voltage measurements are begun on the first day to determine if the internal temperature of the reference is at or near its normal operating temperature. Temperature measurements are not made on succeeding days unless specifically requested by the client The relationship between small day-today temperature fluctuations and output voltage changes is different for each individual reference device and generally is not known; thus the temperature readings are not useful for correcting voltage readings. Voltage measurements are taken once a day for a minumum of 10 d. The data are reviewed at the conclusion of the measurement period and, if the voltages are sufficiently stable, the average of the measurements is reported as the calibrated value.

## 5. Calibration Uncertainties

The uncertainty assigned to the calibrated value of a client standard is a combination of three uncertainties: (1) the uncertainty of assigning a value to the primary cells from the Josephson array, (2) the uncertainty of assigning a value to the working cells from the primary cells each day, and (3) the uncertainty of assigning a value to the client standards considering the NIST measurement uncertainties and the performance of the client standard.

### 5.1 Uncertainty in the Assignment of the Mean Emfs of the Primary Groups

Table 1 summarizes the sources of uncertainty in assigning a value to the mean emf of a primary cell group at the time of a Josephson measurement.

The microwave frequency is measured by a frequency counter which is calibrated in terms of WWVB. An uncertainty is included for measurement uncertainty and drift of the Gunn oscillator frequency.

During a Josephson measurement each Zener reference is compared to the array five times. The pooled standard deviation of the mean calculated from individual comparisons is 0.0042 *μ*V (60 degrees of freedom). This value, divided by the square root of three (i.e., 0.0024 *μ*V, 0.002 ppm), is used as the random component of uncertainty in comparing the mean of the three Zener standards to the Josephson array. In addition, the thermal emfs in the leads to the Zener reference are measured and subtracted from the Zener values. The uncertainty of determining the thermal emfs is 0.0042 *μ*V (0.004 ppm). As a conservative approach we add these two sources of uncertainty directly to obtain a total uncertainty of 0.0066 *μ*V (0.006 ppm). All other known sources of systematic error are negligible.

The random component of uncertainty in comparing the Zener standards to the Primary Group is estimated from the 0.034 *μ*V pooled standard deviation of a single cell-Zener comparison. Because of the redundancy of the measurement design, the uncertainty of the difference of the mean of the Zener reference group to the mean of the Primary Group is 0.012 *μ*V. Two measurement designs are made for the Primary Group (before and after the Josephson measurements); thus, the uncertainty of its mean emf is reduced by the square root of two for a total uncertainty of 0.009 *μ*V (0.009 ppm).

The difference of the mean Zener group emf minus the mean Primary Group emf typically shows a change of (0.008±0.011) *μ*V in the before and after comparisons described above. These appear to be caused by small shifts in the Zener references emfs between the two measurements. Although statistically the bias in the shift (0.008 *μ*V) is not significant compared to 0.11 *μ*V, it is felt that a bias does exist but no correction is made. Rather, an uncertainty of 0.019 ppm (0.008+0.011) is assigned for changes in the mean Zener group emf during the measurements.

Modular low-thermal switches (similar to those described in sec. 2) are used to connect the various primary cells and the three transfer-Zener-standards to the VTP measuring system. The thermal emf variations in these switches that do not cancel on cell reversal have been measured to be 0.005 *μ*V (0.005 ppm).

The root-sum-square (RSS) total is an estimate of the uncertainty in assigning a value to the mean emf of the Primary Group based on one Josephson measurement. This is an estimate of how well the present system would agree with another totally independent Josephson system. Based on data from actual multiple Josephson measurements from our one system we observe a reproducibility of the assignments of the mean emf of a group of standard cells of about 0.020 ppm.

### 5.2 Uncertainty in the Assignment of the Mean Emfs of the Working Groups

Table 2 summarizes the uncertainties in assigning a daily value to the mean emf of one of the working groups.

The uncertainty for the day-to-day fluctuations of the primary groups also includes an additional uncertainty produced by the inexact prediction of the values of the primary groups. The effect of these combined uncertainties has been estimated by an experiment lasting several months where values were assigned to the working groups using different groups of primary cells and comparing the differences in the assignments.

The DVM scale error is estimated based on daily readings of a calibrated 1000 *μ*V source. The random uncertainty of the cell comparisons is estimated from the redundant measurement designs as before with a pooled standard deviation of a single measurement of 0.034 *μ*V. The thermal emf variations in the switches that connect the various cells to measuring system VTP have been measured to be 0.015 *μ*V (0.015 ppm).

The RSS total uncertainty in assigning a value to the mean emf of either working group is thus estimated to be 0.040 ppm.

### 5.3 Uncertainty in the Assignment of the Mean Emf of a Client Cell

Table 3 summarizes the sources of uncertainty in calibrating a client cell in terms of the NIST volt via the regular calibration method (RCS). The uncertainty of the Working-Group assignment is carried over from table 2.

The uncertainty introduced by the within-day changes in the Working-Group was estimated by a several-month-long experiment comparing the Working Group cells to the Primary-Group cells both before and after the normal workload measurements and recording the changes.

The DVM scale error is calculated as described for table 2, except that cell differences as large as 120 *μ*V may be measured. The random measurement uncertainty of the working group—client cell comparison is estimated from the redundant measurement design as before. The thermal emfs in the crossbar scanner are measured periodically as described before and an uncertainty is included for their variations.

The final reported uncertainty for a client-cell emf is the direct sum of three components: (1) the assignment uncertainty from table 3 (0.065 ppm), (2) the uncertainty from the day-to-day cell fluctuations, and (3) the uncertainty caused by the imprecision of the cell temperature measuring device.

The day-to-day random component of the uncertainty of the client-cell emf is based on the standard deviation of the measured cell emfs from the 10 daily measurement designs. This standard deviation is compared to the pooled standard deviation of a large population of measurements of similar standards (0.135—0.188 *μ*V, depending on the type of enclosure) using an F-test at the 99% confidence interval (CI). If the standard deviation is determined to belong to that population, then the population standard deviation of the mean is used as the estimate of the random component. If not, the computed standard deviation of the mean is used as the estimate.

The uncertainty of the assignment of values to the cell emfs due to the imprecision of the temperature monitoring device is estimated by calculating the change in cell emf (according to the International Temperature formula) for a change of one least count of the temperature monitoring device. For cells in enclosures containing internal thermistor bridges, where a least count of 0.001 °C is possible, an additional uncertainty of approximately 0.05 ppm is included. Enclosures monitored by mercury-in-glass thermometers are assigned an uncertainty equivalent to half the smallest graduation marked on the thermometer, approximately 0.5 ppm for a resolution of 0.01 °C. For cells calibrated in NIST oil baths an emf uncertainty equivalent to a temperature uncertainty of 0.005 °C is used to account for possible calibration errors in the platinum resistance thermometers used at NIST.

The final reported uncertainty contains no allowance for long term drift of the cells under test. Long term behavior must be determined by the client by analysis of the history of each individual standard. In addition, no allowances are made for the possible effects of transporting the standard between laboratories or the possible existence of a gross temperature dependence on ambient (room) temperature of the standard.

### 5.4 Uncertainties in the Determination of V(LAB)-V(NIST)

Table 4 summarizes the sources of uncertainty in the determination of V(LAB)-V(NIST) obtained via the Volt Transfer Program.

The uncertainty in assigning a value to the transport standard while at NIST is obtained through an analysis identical to table 3, except the switches used for the VTP transport standards have some- what lower uncompensated thermal emfs (0.020 *μ*V as opposed to the 0.030 *μ*V shown in table 3).

The transport standards used for the Volt Transfer Program all contain thermistor bridges with 0.001 °C resolution; an uncertainty for the cell emf equivalent to this temperature change is included. In addition, the cell temperature within the enclosures changes slightly with changes in ambient temperature. The thermistor bridge does not properly reflect the change of the temperature of the cell in this case, probably due to a temperature sensitive component of the bridge circuitry that is at ambient temperature. Based on experiments on similar enclosures, an uncertainty estimate of 0.05 ppm is included to account for a difference between the client laboratory ambient temperature and the NIST laboratory ambient temperature.

Each of the four cells in the transport standard is used to determine a value for the difference in laboratory units, V(LAB)-V(NIST). The standard deviation of these four values arises from the uncertainty in predicting the cell emfs while at the client laboratory, the uncertainty in comparing the client laboratory reference to the transport standard, and the uncertainty due to random changes in the cell emfs due to shipment. A pooled standard deviation of 0.19 *μ*V has been computed from 50 transfers and is used as the population standard deviation. The calculated standard deviation for each new transfer is compared to the population standard deviation at the 99% confidence interval using an F-test. If the statistic is determined to belong to that population, then the population standard deviation of the mean is used as the uncertainty estimate; if not, the actual standard deviation of the mean is used. Thus the uncertainty estimate for a four cell transport standard, based on the population standard deviation, is $(0.19\;\mu V)/\sqrt{4}$ or 0.095 μV (0.093 ppm).

If the difference V(LAB)-V(NIST) exceeds 0.14 ppm (≈ 1.5 times the uncertainty based on the population standard deviation), the report will recommend adjusting the assigned values of the client reference cell emfs to reduce the difference to zero. Adjusted values for the client cell emfs are calculated based on the measurements made in the client laboratory comparing the client reference group to the NIST transport standard. Each comparison results in a determination of the difference of each client reference cell emf from the mean emf of the client reference group. The average difference for each cell, obtained from all the comparisons, is added to the newly determined mean emf of the reference group to calculate the new values for each of the client reference cells.

### 5.5 Uncertainty in the Calibration of Solid-State Standards

The ZCS measuring system was designed principally to measure voltage standards in the range of 5 to 10 V. Table 5 lists the sources of uncertainty in the measuring system extrapolated to the worst case unknown voltage in the 5 to 10 V range. The system may be used over the 1 to 5 V range but with somewhat reduced accuracy except when the unknown voltage is near a cardinal value (1.018, 2.036, 3.054,… V).

By far the most critical component of the system is the digital voltmeter. The DVM gain of the 1-V range is measured during the course of the Zener measurements and the gain error is calculated and applied as a correction to the appropriate DVM readings. An allowance is included in table 5 for the inaccuracy of this gain measurement.

The DVM linearity was initially checked on the 10-V range using a calibrated, manual 7-dial Kelvin-Varley divider, and measurements on similar DVMs indicate little or no change in the linearity with time. The linearity error is typically a maximum of 0.7 ppm or less at half-scale input and no correction to the data is made for it Provision has been made for the system to calibrate automatically the linearity of the DVM at 10 points on the 10-V range by measuring the 10 voltages 1.018, 2.036,…, 10.18 V, although this has not been necessary. Estimates of the uncertainties for the other sources of error are calculated as described previously.

## 6. Quality Control Procedures

Several additional quality control procedures are performed periodically to estimate or eliminate potential sources of error in the measurement systems that may go undetected by the redundant measurement designs. These procedures include measuring the scaling or gain errors in the digital voltmeters, the uncompensated thermal emfs in the crossbar switches and input leads, the leakage currents to ground from the measurement apparatus, and the circulating ground currents.

Before each daily set of cell comparisons each cell measurement system reads the output of its own 1000 *μ*V Zener source to monitor the gain error of the 0.1 V range of the digital voltmeter. All voltmeter measurements are taken with the applied voltage in the normal polarity and again with the polarity reversed by the crossbar switch to eliminate any uncertainty due to zero offset of the voltmeter and thermal emfs in the leads to the voltmeter. The daily measured values of the 1000 *μ*V source are plotted on a control chart and compared to predetermined limits. The gain error of the DVM in the solid-state measurement system (ZCS) is evaluated and corrected at the time of the measurement as described in section 2.

Thermal emfs in the leads from the client standards to the crossbar switch are evaluated regularly. The positive and negative leads are shorted together at the end where they would normally connect to the standard and a redundant measurement design is made between two sets of shorted leads. These thermal emf measurements are performed frequently, usually every 2 months or so as cell enclosures leave the laboratory and leads become free. Experience has shown that the thermal emfs in individual switches are usually small and fairly constant (typically less than 15 and 30 nV for measuring systems VTP and REG, respectively).

A standard cell group that can be used exclusively as a long term check standard is not available because of a shortage of good quality temperature regulated enclosures and long-term stable cells. Instead, the daily change in the average emf of the cell workload is calculated and compared to control limits. For the solid-state measuring system, a 10-V standard that is not moved is measured every day as part of the workload and serves as a check standard to monitor the long term stability of the measurement system.

Additional tests are also occasionally performed on the automated cell systems. Measurements of the insulation/leakage resistance of the measuring systems are done and recorded in laboratory notebooks. Closure experiments are done monthly (at least) to detect systematic errors due to leakage resistance. Closure experiments consist of redundantly comparing three cell enclosures with the pattern, A-B, B-C, and C-A. Summing the three mean emf differences should yield a value of zero; the deviation of the actual value from zero is an indication of measurement error. Using 24 such experiments performed on different enclosures between July 17, 1986 and October 1, 1986, the mean closure error was determined to be —0.9 nV with a standard deviation of the mean of 1.7 nV.

## 7. Conclusions

We are currently calibrating about 400 voltage standards per year. Although the measurement of the standards and the initial data reduction are nearly fully automated, the final analysis of the data and the generation of test reports still requires considerable attention from NIST laboratory personnel. We are presently in the process of developing advanced data handling and reduction tools (i.e., computer programs) to minimize the staff time required to generate the test reports.

## Figures and Tables

**Table 1:** Uncertainties in the assignment of the mean emfs of the primary group

Source of uncertainty	1 std. dev. estimate (ppm)
Microwave frequency	0.005
Assignment of Zener reference group using array	0.006
Random uncertainty of comparison of Zener reference group to the mean of the Primary Group	0.009
Change in the Zener reference group during the Josephson measurement	0.019
Uncompensated thermal emfs in the cell switches	0.005
RSS total	0.023

**Table 2:** Uncertainties in the assignment of the mean emfs of a working group

Source of uncertainty	1 std. dev. estimate (ppm)
Assignment of Primary Group (from table 1)	0.023
Day-to-day fluctuation of Primary Group	0.028
DVM scale error	0.005
Random uncertainty in comparison of Primary Group to Working Group	0.004
Uncompensated thermal emfs in cell switches	0.015
Uncertainty of assignment to Working Group	
RSS total	0.040

**Table 3:** Uncertainties in the assignment of the mean emf of a client cell

Source of uncertainty	1 std.dev. estimate (ppm)
Uncertainty of assignment to Working Group (from table 2)	0.040
Change in Working Group during the day	0.040
DVM scale error	0.010
Random uncertainty of comparison of the Working Group to client cell	0.007
Uncompensated thermal emfs in the cell switches	0.030
Uncertainty of assignment to client cell (not including temperature measurement errors and day-to-day client cell emf fluctuations; see text)	
RSS total	0.065

**Table 4:** Uncertainties in the determination of V(LAB)-V(NBS)

Source of uncertainty	1 std.dev. estimate (ppm)
Uncertainty of NIST assignment to transport group	0.061
Correlated temperature effects of the transport	0.050
Temperature resolution	0.050
Random component due to individual cell assignments and changes during transport	0.093
Uncertainty of V(LAB)-V(NIST) determination using a four cell transport enclosure	
RSS total	0.132

**Table 5:** Uncertainties in the assignment of the mean emf of a solid-state standard

Source of uncertainty	1 std.dev. estimate (ppm)
DVM gain uncertainty	0.020
DVM linearity uncertainty	0.062
DVM leakage/bias currents	0.023
Standard cell leakage currents	0.006
Scanner switch thermal emfs	0.013
Random uncertainty in calibrating the Transfer Reference	0.007
Random uncertainty in calibrating the client standard (within-day)	0.013
RSS subtotal	0.072
Uncertainty in value of the Working Group	0.040
Change in Working Group during the day	0.047
Uncertainty of the value assigned to the client standard	
RSS Total	0.095

## Footnotes

1 The dominant system of units used throughout the world to express the results of physical measurements is Le Système International d’Unites or International System of Units, abbreviated SI. The Consultative Committee on Electricity (CCE) of the International Committee of Weights and Measures believes that the appearance of creating of new unit system outside of the SI must be avoided. Thus, all “practical units of voltage” are to be viewed as representations of the (SI) volt. Further, the CCE believes that the representations of the volt of national standards laboratories are sufficiently close to the Si volt that it will rarely be necessary to distinguish between them and the SI volt. However, for clarity within this paper, the representation of the volt as maintained at NIST will be denoted as the NIST volt or V(NIST).
